# Supplementary material for: Precise visuomotor transformations underlying collective behavior in larval zebrafish
Source: Nat Commun. 2021 Nov 12;12:6578. doi: 10.1038/s41467-021-26748-0 (PMC8590009; doi:10.1038/s41467-021-26748-0)
Supplement: Supplementary file 1 — Supplementary Information [file 41467_2021_26748_MOESM1_ESM.pdf]

Supplementary information for:

Precise visuomotor transformations underlying collective behavior in larval zebrafish

Roy Harpaz[1,2], Minh Nguyet Nguyen[3], Armin Bahl[4], Florian Engert[1,2]

This file contains supplementary figure S1-S7

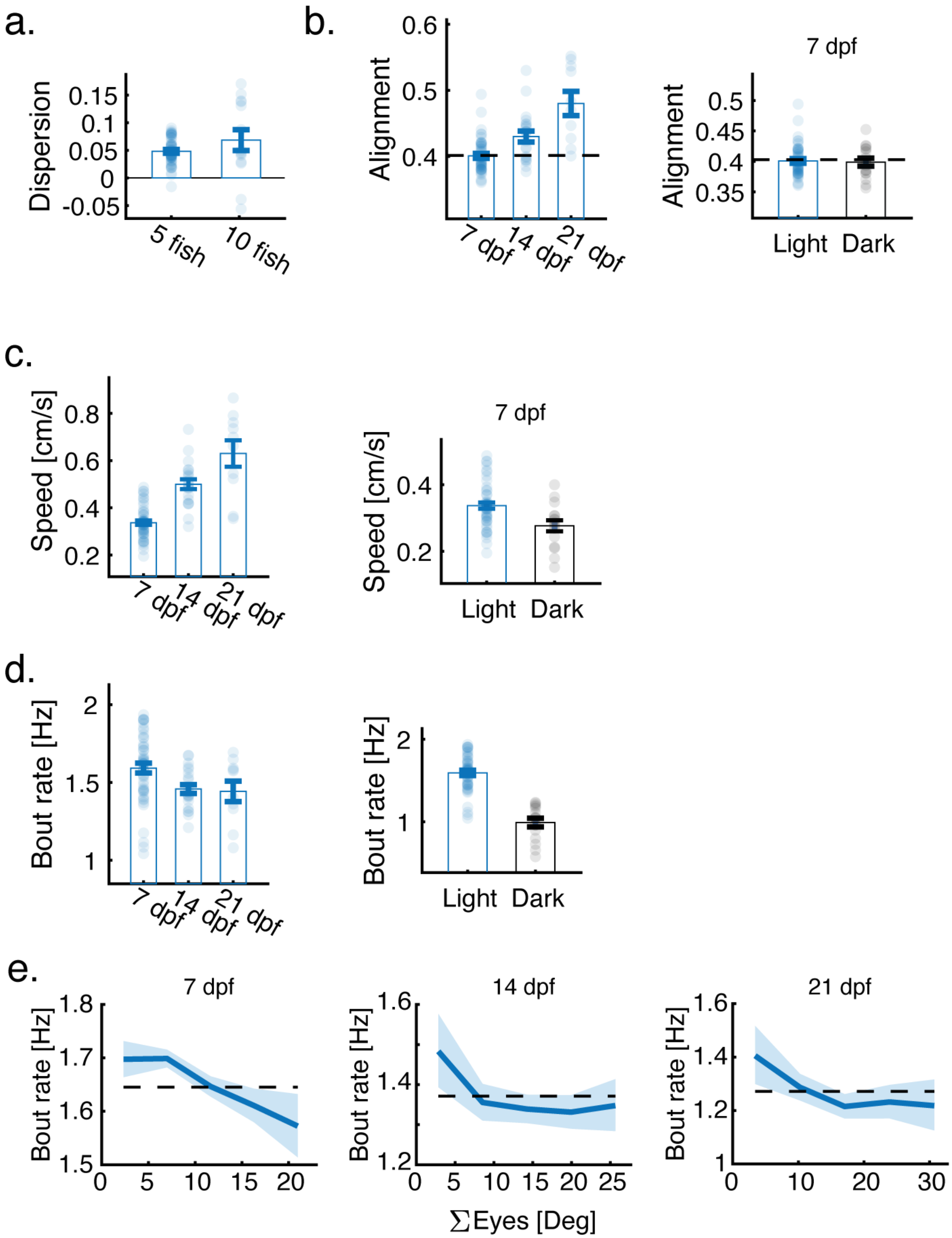

a.
Dispersion
0.15
0.1
0.05
0
-0.05
5 fish
10 fish
b.
Alignment
0.6
0.5
0.4
7 dpf
14 dpf
21 dpf
7 dpf
Alignment
0.5
0.45
0.4
0.35
Light
Dark
c.
Speed [cm/s]
0.8
0.6
0.4
0.2
7 dpf
14 dpf
21 dpf
7 dpf
Speed [cm/s]
0.4
0.2
Light
Dark
d.
Bout rate [Hz]
2
1.5
1
7 dpf
14 dpf
21 dpf
Bout rate [Hz]
2
1
Light
Dark
e.
7 dpf
Bout rate [Hz]
1.8
1.7
1.6
1.5
0
5
10
15
20
14 dpf
Bout rate [Hz]
1.6
1.4
1.2
0
5
10
15
20
25
21 dpf
Bout rate [Hz]
1.6
1.4
1.2
1
0
10
20
30
∑Eyes [Deg]

Figure S1: Visual social interactions develop with age. a. Groups of 5 and 10 fish at age 7 dpf are more dispersed than chance levels ($P_5=8 \cdot 10^{-10}$, $N_5=48$; $P_{10} = 0.003$, $N_{10} = 14$, two sided t-test; $t_5 = 7.7$, $t_{10} = 3.64$) **b.** Average alignment of free-swimming groups at different ages (left), and at age 7 dpf for fish swimming in the light and in the dark (right). Group alignment increases over development ($P=10^{-7}$, one-way ANOVA, $F=23$; Same groups as in Fig. 1c-d). Bars represent mean over groups and error bars are SEM; dotted lines are group polarity values calculated for shuffled groups (Methods). **c.** Same as in b, but showing the average speed of groups. Speed increases with age ($P=6 \cdot 10^{-15}$, one-way ANOVA, $F = 51$) and is decreased when 7 dpf fish swim in the dark (Right, P=0.0025, two sided t-test, $t = 3.15$). **d.** Same as in b but showing the bout rate of the fish. Bout rate decreases at age 14 and 21 dpf compared to 7 dpf (Left, $P_{14\ vs\ 7}=0.011$, $P_{21\ vs\ 7}= 0.053$, two-sided t-test, $t_{14\ vs\ 7} = 2.6$, $t_{21\ vs\ 7} = 1.97$), and also when 7 dpf fish swim in the dark (Right, $P=6 \cdot 10^{-14}$, two-sided t-test, $t=9.6$). **e.** Bout rate of the fish as a function of the total retinal occupancy experienced on both eyes. Fish of all ages tend to reduce their bout rate when they experience high visual occupancy. Bold lines represent the bout rate calculated from bout events recorded from all fish in 5° bins; error bars are the 95% confidence interval of the Binomial distribution fitted to the observed bout probability in each bin.

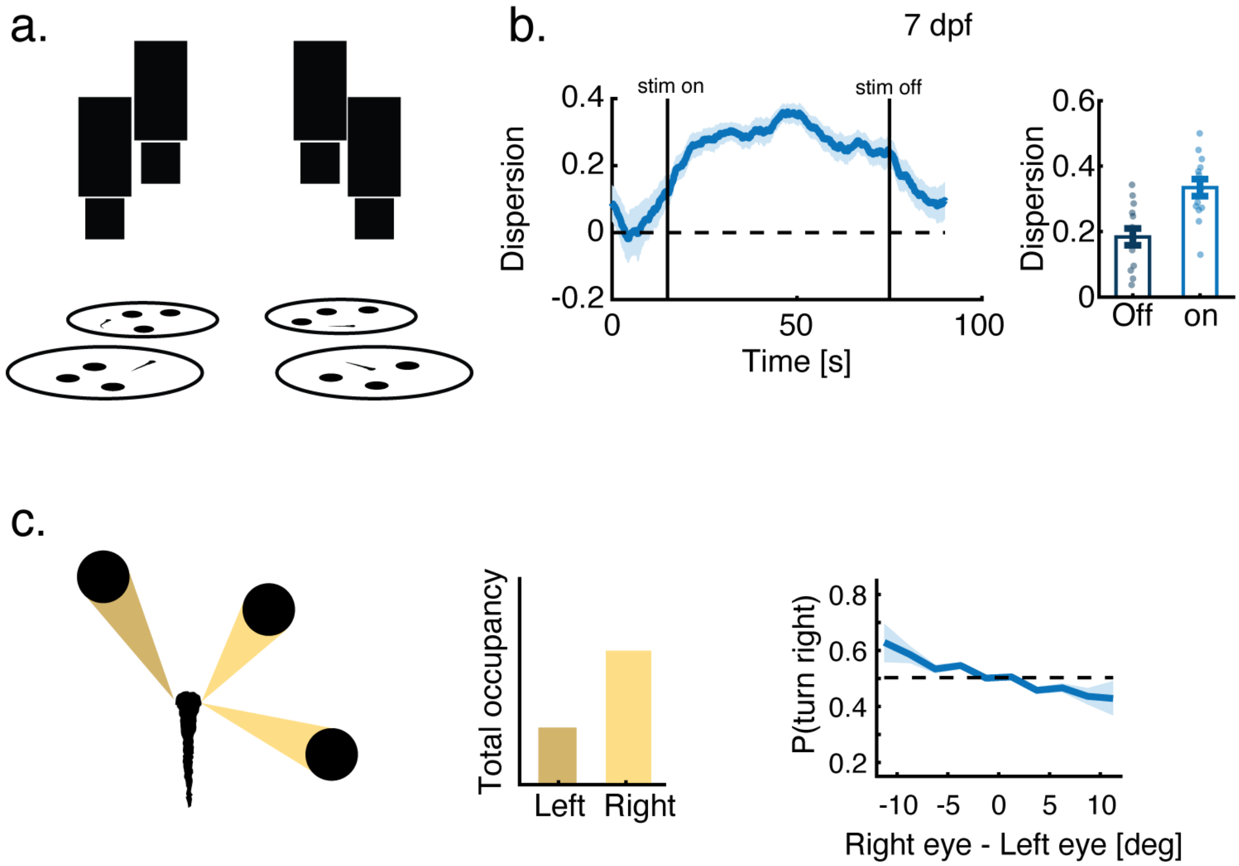

Figure S2: Virtual reality captures the structure and interactions of real groups at 7 dpf. a. Sketch of the experimental assay in which 4 fish swimming individually in different arenas are combined together via bottom projected dots mimicking the motion of real fish in separate arenas (1)(Methods). **b.** Left: Dispersion of the virtual group increases significantly when dots mimicking neighboring fish are visible to the real fish ('stim on') compared to when they are turned off. Blue line is the average over groups and trials (N = 14 groups, with 60 trials each); shaded area is SEM. Right: Dispersion values are averaged over all times when stimulus is on vs off ($P=10^{-6}$, N = 14 groups; two sided within subjects t-test, $t =- 8.01$). **c.** Left: Sketch showing the total angular occupancy that projected dots occupy on each of the eyes of a focal fish. Right: Probability to turn right as a function of the difference in total angular occupancy experienced by each eye (negative values - higher occupancy to the left). Bold lines represent turning probability calculated as the fraction of right turns out of all turns collected from all fish in 5° bins; error bars are the 95% confidence interval of the fitted Binomial distribution to the events in each bin. At 7 dpf, larvae tend to turn away from the more occupied side, similar to the responses observed in group swimming experiments (Compare to Fig. 1f top left).

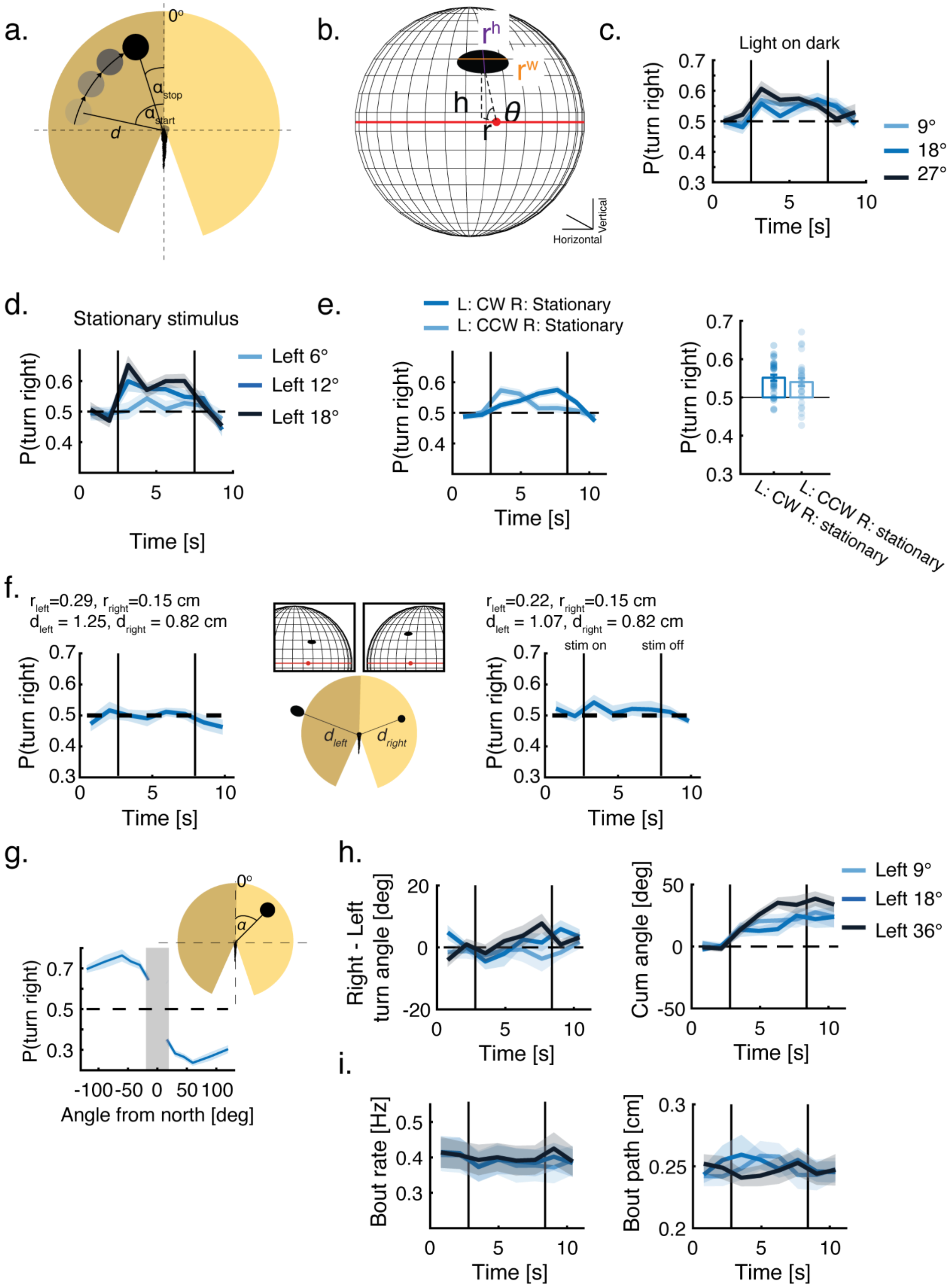

a.
0°
α stop
α start
d
b.
r^h
r^w
h
θ
r
Vertical
Horizontal
c.
Light on dark
P(turn right)
Time [s]
9°
18°
27°
d.
Stationary stimulus
Left 6°
Left 12°
Left 18°
P(turn right)
Time [s]
e.
L: CW R: Stationary
L: CCW R: Stationary
P(turn right)
Time [s]
L: CW R: stationary
L: CCW R: stationary
f.
r_left=0.29, r_right=0.15 cm
d_left = 1.25, d_right = 0.82 cm
d_left
d_right
r_left=0.22, r_right=0.15 cm
d_left = 1.07, d_right = 0.82 cm
stim on
stim off
P(turn right)
Time [s]
g.
0°
α
P(turn right)
Angle from north [deg]
h.
Right - Left turn angle [deg]
Cum angle [deg]
Time [s]
Left 9°
Left 18°
Left 36°
i.
Bout rate [Hz]
Bout path [cm]
Time [s]

Figure S3: VR reveals the algorithms young larvae use to integrate visual social information. **a.** Sketch of a virtual dot moving tangentially around the fish. In every trial, an image (or images) of a given size and shape appear at a starting angle α_{start} and at distance d from the fish. The image moves in pancuated bouts mimicking fish motion, at a constant distance d, to its stopping angle $\alpha_{stop}$and disappears (Methods)(Movies 6-7). **b.** Sketch of the image of a dot on the retina and its calculated properties (Methods). Red dot is the center of the back of the retina, and the red line represents the horizon line. r^{h}- is the height (or vertical length) of the image, r^{w}- is the width (or horizontal length) of the image, h, r, θ are the polar coordinates of the center of the image with respect to the center of the retina. **c.** Probability to turn right per bout when a single moving light dot of different sizes is presented on a dark background to the left of the fish (compare to Fig. 2c) (N=32 fish). **d.** Probability to turn right per bout for stationary dots at different distances, presented to the left of the fish (N=16 fish). **e.** Left: Probability to turn right per bout when a moving dot of 9° is presented to the left eye, moving either in clockwise or counterclockwise direction, and a stationary dot of the same size is presented to the right eye (N=32 fish). Right: Probability to turn right per bout averaged over the entire trial duration. Bars represent means and error bars represent SEM, same data as shown on the left. **f.** Left: Probability to turn right per bout when two images of different sizes r_{left}, r_{right}(representing the major axis of the plotted ellipse) at distances d_{left}, d_{right} are presented on both sides of the fish resulting in similar visual occupancy on both retinae, yet at different positions (N=32 fish). Inset shows the projected images, sizes and distances and their retinal images (all sizes are to scale). Similar occupancy on the retinae results in an equal likelihood to turn in either direction. Right: same but for a different set of sizes and distances. **g.** Probability to turn right when a stationary dot is presented at different angles (and at a constant distance) with respect to the heading of the fish (0°). Positive angles are to the right of the fish. Gray shaded area represents the expected binocular zone in the visual field, which we did not stimulate. **h.** Difference in mean turning angle between rightward and leftward turns (left) and cumulative turning angle (right), when dots of different sizes are presented to the left of the fish (N=24 fish). **i.** Mean bout rate (left) and path traveled during a bout (right), when dots of different sizes are presented to the left of the fish (N=24 fish). In panels c-g, probability to turn right per bout is calculated as the fraction of right turns out of all turns in 1.25s time bins (Methods). In panels c-i bold line represents average over fish; shaded area represents SEM.

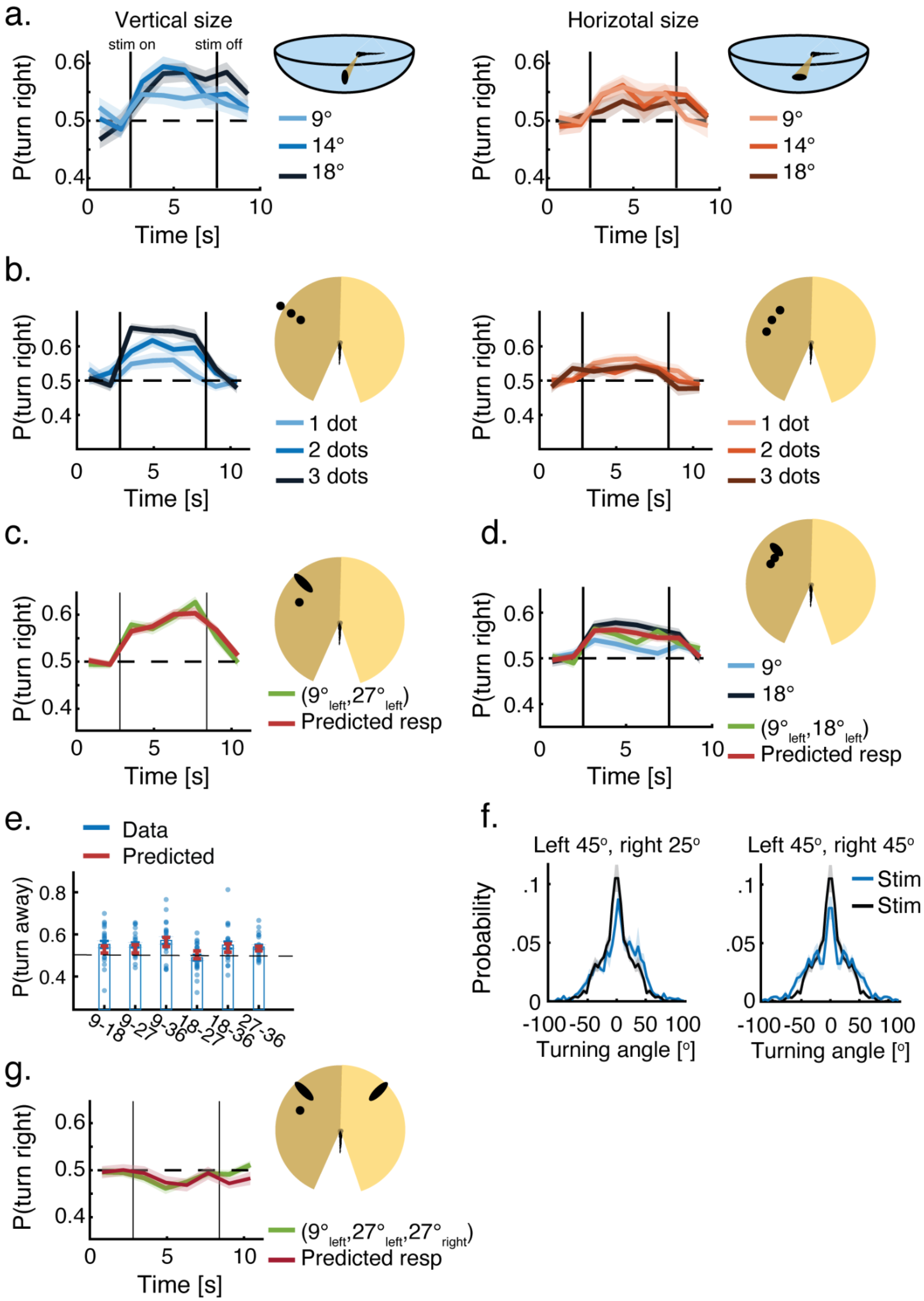

a.
Vertical size
stim on
stim off
P(turn right)
0.6
0.5
0.4
0
5
10
Time [s]
9°
14°
18°
Horizotal size
b.
1 dot
2 dots
3 dots
c.
(9°left, 27°left)
Predicted resp
d.
9°
18°
(9°left, 18°left)
Predicted resp
e.
Data
Predicted
P(turn away)
0.8
0.6
0.4
9-18
9-27
9-36
18-27
18-36
27-36
f.
Left 45°, right 25°
Left 45°, right 45°
Stim on
Stim off
Probability
.1
.05
0
-100
-50
0
50
100
Turning angle [°]
g.
(9°left, 27°left, 27°right)
Predicted resp

Figure S4: VR reveals how 7 dpf larvae integrate information from multiple neighbors. a. Left: Probability to turn right per bout when ellipses of increasing vertical size (perpendicular to the plane of the eye) are presented on the sidewall of a half dome shaped arena, while horizontal sizes remain constant at 9° (N=32 fish, Methods). Right: Same as on the left, only for ellipses of increasing horizontal sizes (parallel to the plane of the eye), while vertical sizes remain constant at 9° (N=32 fish). Results are similar to those observed for ellipses presented on the bottom of the tank (Fig. 2d). **b.** Left: Probability to turn right per bout when one, two or three dots of similar size (9°) are presented to the left visual field. Dots are positioned at the same angle from the fish, but with different radial distances (N = 32). Right: same only for dots that are positioned at different angles from the fish and at a similar radial distance (N = 32). Probability to turn right increases with the increase in the occupancy in the vertical dimension but not in the horizontal one (same as Fig. 2d for ellipses of different sizes). **c.** Similar to Fig. 2e but with a different combination of stimuli (N = 32 fish). **d.** Similar to Fig. 2e, but with no space between the two images presented to the left of the fish. Larvae respond in a similar manner when the two stimuli are separated from one another and when they are not (N=32 fish). **e.** Mean probability to turn right when two dots of different sizes are presented to both eyes of the fish (in this plot, the larger dot is presented to the left visual field, Methods). Bars represent mean over fish (N = 24; blue dots); error bars represent SEM. Overlaid are the mean and the SEM of the predicted responses of the groups based on the linear summation of the observed response biases elicited by each stimulus presented alone (red bars)(see Fig. 2f. **f.** Distribution of fish turning angles when two stimuli of different sizes (left) and of similar sizes (right) are presented on both sides of the fish (blue). For comparison, the distribution of fish turning angles without any stimuli is also shown (black). Turning angles for each fish (N=8 fish) are grouped into 5° bins over all trials; bold lines represent mean over fish and shaded areas are SEM. The distributions of turning angles indicate that fish do not average stimuli sizes, but rather probabilistically respond to one of the two presented stimuli in each bout. **g.** Probability to turn right per bout when two images are presented to the left eye and a single image is presented to the right one (green line)(N = 32 fish). The response to the joint presentation of the stimuli is accurately predicted by averaging the responses obtained when the images are presented alone within an eye and by the linear summation of the averaged response biases between the eyes (red line; eq. 1, Methods). N = 32 fish. In panels a-e and g, probability to turn right per bout is calculated as the fraction of right turns out of all turns in 1.25s time bins (Methods). In all panels, bold lines are averages over fish and shaded areas are SEM.

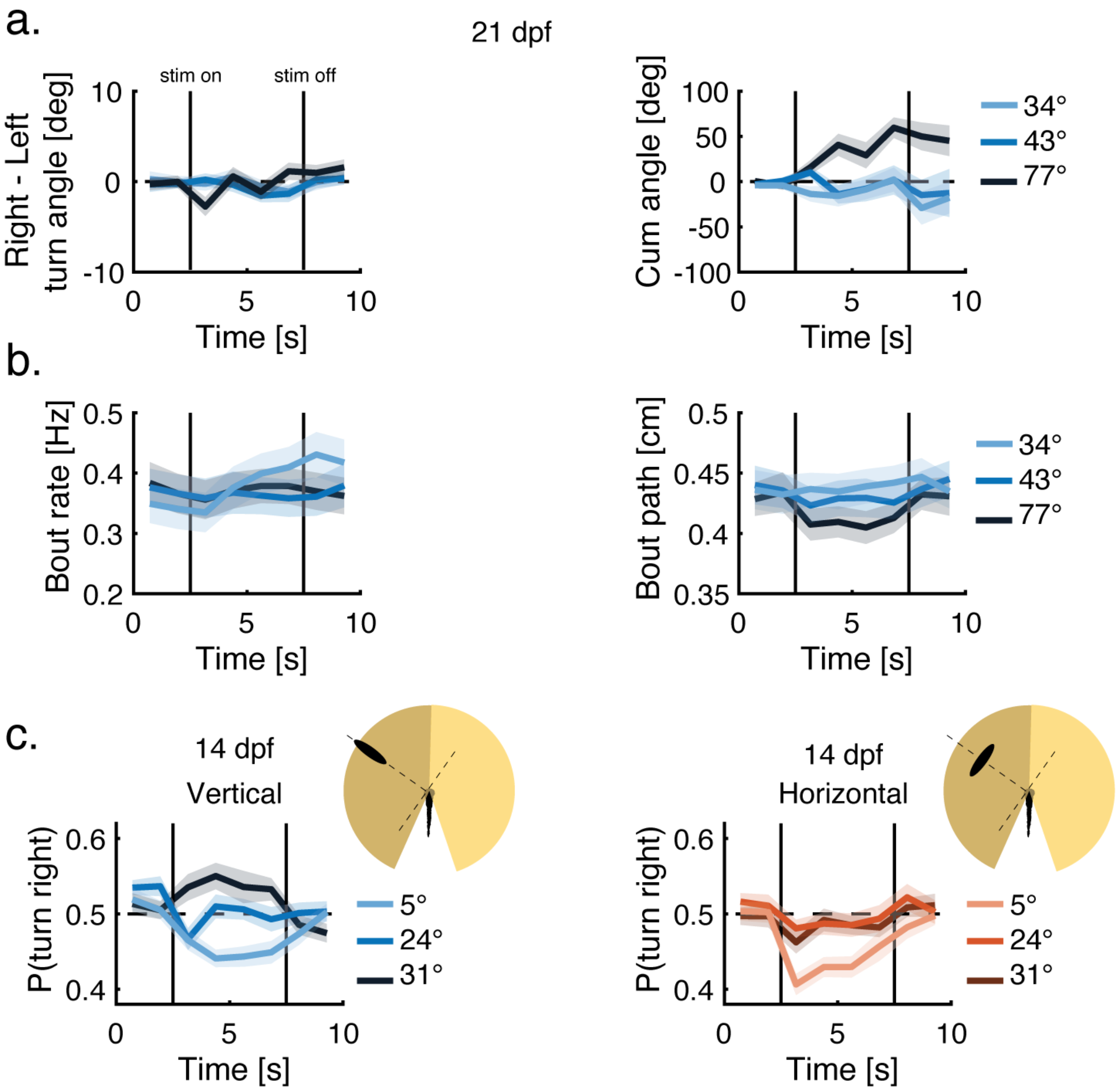

Figure S5: VR reveals that older larvae use similar algorithms to those seen in young larvae. a. Difference in mean turning angle between rightward and leftward turns (left) and cumulative turning angles (right), when images of different angular sizes are presented to the left of 21 dpf fish (Methods). Vertical lines represent times when stimuli are turned on and off (N=32 fish). **b.** Mean bout rate (left) and path traveled during a bout (right), when images of different angular sizes are presented to the left of 21 dpf fish (N=32 fish). **c.** Left: Probability to turn right per bout in response to ellipses of increasing vertical size (perpendicular to the plane of the eye), while horizontal sizes remain constant at 9º. (N=32 fish, age 14 dpf). Right: Probability to turn right in response to ellipses of increasing horizontal sizes (parallel to the plane of the eye), while vertical sizes remain constant at 9º (N=32 fish, age 14 dpf). Probability to turn right per bout is calculated as the fraction of right turns out of all turns in 1.25s time bins (Methods). In all panels, bold lines are averages over fish and shaded areas are SEM.

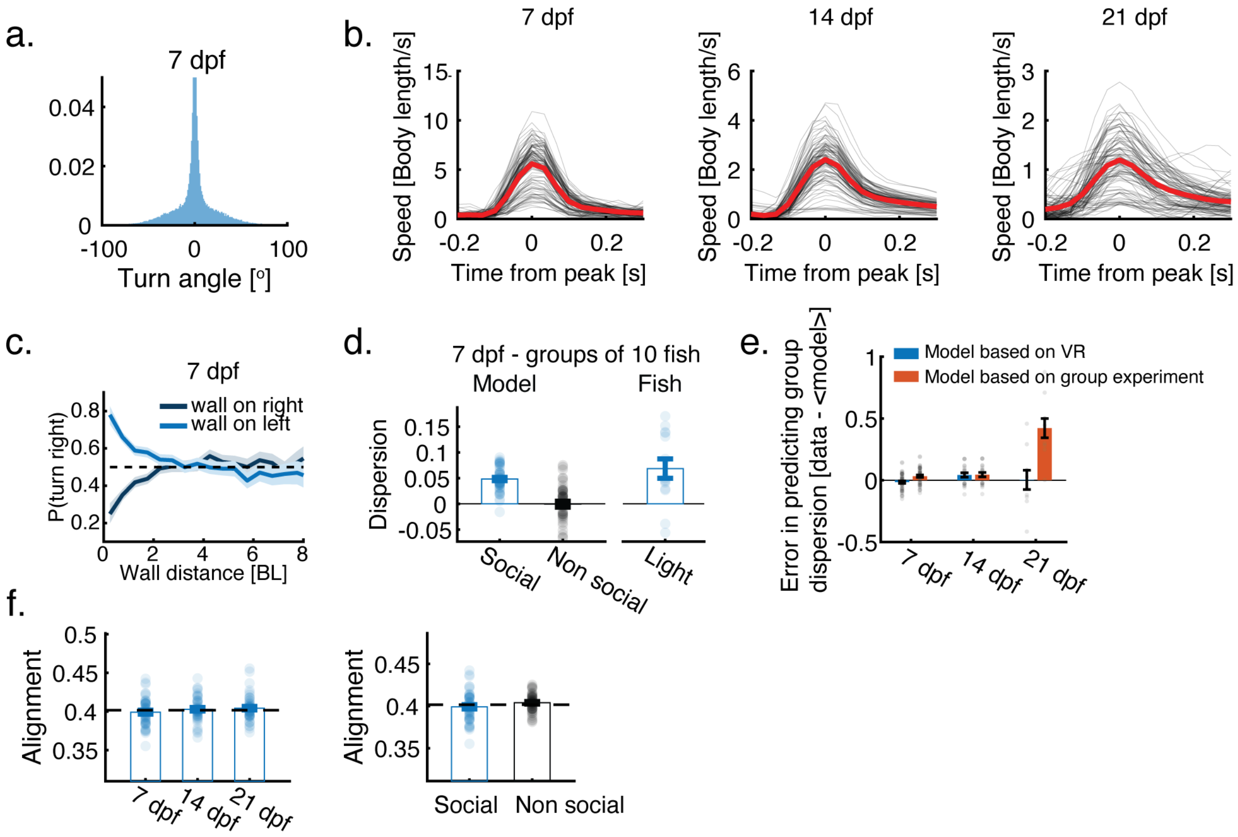

Figure S6: Modeling collective behavior based on responses to visual occupancy from VR and swimming statistics of real fish. a. Distribution of turning angles estimated from all experiments of 7 dpf fish swimming in the light (N = 48 groups of 5 fish). **b.** Speed profiles of real fish at different ages (black) and the average observed profile (red)(averages are based on 100 bouts extracted from the different age groups). Speed profiles in the simulations were taken to approximate the average path travelled in a bout for the different ages (Methods). **c.** Probability to turn right as a function of the distance and direction (left/right) to the closest wall. Bold line is the probability estimated from right/left turns collected from all fish in 1 body length bins; shaded area is the 95% confidence interval of the fitted Binomial distribution to the events in each bin. Fish consistently turn away when swimming close (< 3 body lengths) to a wall. **d.** Simulated groups of 7 dpf fish (10 fish in a group) with social interactions are more dispersed than is expected by chance and are also more dispersed than simulated groups without social interactions. Bars represent means; error bars are SEM (N=50 simulations). Experimental data of groups of 10 fish swimming in the light is plotted for comparison (N = 14). **e.** Mean error in predicting the average dispersion of real groups for models that are based on social interactions extracted from the VR assay (blue, Fig. 4a) and for models that are based on the interactions inferred directly from group swimming experiments (red, Fig. 1f). Individual points represent the distance of the dispersion value of each real group from the average of the model; Error bars represent SEM. **f.** Mean polarity values for simulated groups of fish with social interactions for different age groups (left) and for simulated 7 dpf fish with and without social interactions(right). Bars represent means; error bars are SEM. Alignment values in all simulations are at chance levels (dotted black lines, Methods).

a.

b.

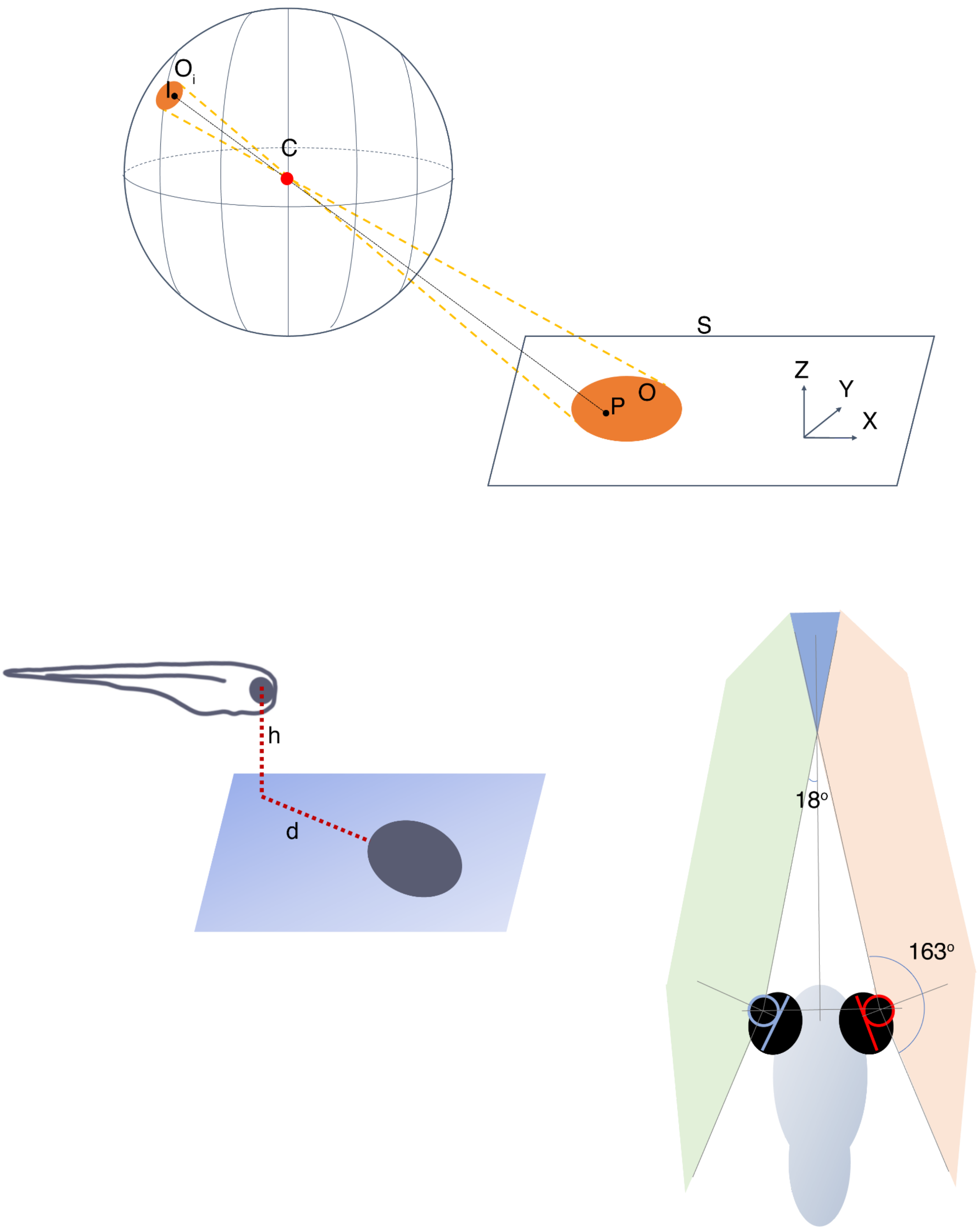

Figure S7: Schematics of retina model. a. To estimate the retinal properties of the projected stimuli presented to the fish we used a pinhole model of the retina in which S is the plane of the projection

screen; O is the projected object onto the screen; P is a point in O; O_I is the image of object O on the modeled retina; I is the image of point P on the modeled retina; C is the center of the eye, or the pinhole that allows light into the retina. The projected 3D position I of point P from the plane S is given by: $I = (C - P) \cdot \frac{r^{eye}}{|\vec{PC}|} + C$

Where $I, C, P \in R^3$, |.| is the euclidean norm and r^{eye} is the radius of the sphere of the eye. After we trace every point P in O to I, we obtain the resulting image O_I on the modeled retina. We then determine the height, width and position of O_I on the retina. **b.** A sketch depicting the height (h = 5mm) and distance (d) of the projected image from the eye of the fish (left) and the distance between the eyes (1.2mm), radius of the eye (0.45mm), effective retina field (163°) and mean vergence angle (36°) we used in our model.

Supplementary References

1. J. Larsch, H. Baier, Biological Motion as an Innate Perceptual Mechanism Driving Social Affiliation. *Curr. Biol.* **28**, 3523-3532.e4 (2018).
