# Supplementary material for: Precise visuomotor transformations underlying collective behavior in larval zebrafish
Source: Nat Commun. 2021 Nov 12;12:6578. doi: 10.1038/s41467-021-26748-0 (PMC8590009; doi:10.1038/s41467-021-26748-0)
Supplement: Supplementary file 3 — Description of Additional Supplementary Files [file 41467_2021_26748_MOESM3_ESM.docx]

**Description of Additional Supplementary Files**

**Title: Supplementary Movie 1-3: Free swimming behavior of larvae in a group.**

**Description:** Example groups of 5 larvae swimming together at ages 7 dpf (Movie 1), 14 dpf (Movie 2) and 21 dpf (Movie 3). Colors represent individual fish; movies are shown at x2 real speed.

**Title: Supplementary Movie 4: Estimating retinal occupancy using ray casting.**

**Description:** Example showing the estimated visual angle that each neighbor occupies on the eye of a 7 dpf focal fish (right), together with total angular occupancy experienced on each eye (left). Yellow cones represent angular sizes of neighbors on the right eye and brown cones on the left eye. Movie is shown at x5 real speed.

**Title: Supplementary Movie 5: Estimating projected retinal images using a pinhole model of the retina. Description:** Examples showing two projected dots, sizes 36^o^ and 9^o^, at 4 distinct angles around the head of the fish from $\pm$60 to $\pm$30 (the eyes of the fish and the planes of the retinea are also shown)(left) and their corresponding retinal images (right). Red dots represent the centers at the back of the retinae.

**Title: Supplementary Movie 6: Fish responding to a closed-loop monocular stimulus.**

**Description:** Example trial of a fish responding to a dot of size 36^o^ moving in intermittent bouts tangentially to its right (from 60^o^ to 30^o^, where 0 is the fish’s heading direction). Trials begin with 2.8s without a stimulus, followed by 5.6s of stimulus presentation and end with an additional 2.8s without a stimulus. Stimulus is presented to the fish only at times when the fish is stationary.

**Title: Supplementary Movie 7: Fish responding to closed-loop binocular stimuli.**

**Description:** Example trial of a fish responding to two dots, sizes 9^o^ (left) and 36^o^ (right), moving in intermittent bouts tangentially around its head (from $\pm$60^o^ to $\pm$30^o^, where 0 is the fish’s heading direction). Trials begin with 2.8s without stimuli, followed by 5.6s of stimuli presentation and end with an additional 2.8s without stimuli. Stimuli are presented to the fish only at times when the fish is stationary.

**Title: Supplementary Movie 8-10: Simulated groups based on algorithms extracted from VR.**

**Description:** Examples of 5 simulated fish swimming in groups at ages 7 dpf (Movie 8), 14 dpf (Movie 9) and 21 dpf (Movie 10). Simulated fish interact with one another based on the algorithms extracted from VR experiments (Fig. 4A and Methods). Colors represent individual fish
