# Supplementary material for: Precise visuomotor transformations underlying collective behavior in larval zebrafish
Source: Nat Commun. 2021 Nov 12;12:6578. doi: 10.1038/s41467-021-26748-0 (PMC8590009; doi:10.1038/s41467-021-26748-0)
Supplement: Supplementary file 14 — Reporting Summary [file 41467_2021_26748_MOESM14_ESM.pdf]

nature research

Corresponding author(s): Roy Harpaz

Last updated by author(s): Oct 1, 2021

# Reporting Summary

Nature Research wishes to improve the reproducibility of the work that we publish. This form provides structure for consistency and transparency in reporting. For further information on Nature Research policies, see our Editorial Policies and the Editorial Policy Checklist.

## Statistics

For all statistical analyses, confirm that the following items are present in the figure legend, table legend, main text, or Methods section.

| n/a | Confirmed | |
|---|---|---|
| ☐ | ☒ | The exact sample size ($n$) for each experimental group/condition, given as a discrete number and unit of measurement |
| ☐ | ☒ | A statement on whether measurements were taken from distinct samples or whether the same sample was measured repeatedly |
| ☐ | ☒ | The statistical test(s) used AND whether they are one- or two-sided<br>*Only common tests should be described solely by name; describe more complex techniques in the Methods section.* |
| ☐ | ☒ | A description of all covariates tested |
| ☐ | ☒ | A description of any assumptions or corrections, such as tests of normality and adjustment for multiple comparisons |
| ☐ | ☒ | A full description of the statistical parameters including central tendency (e.g. means) or other basic estimates (e.g. regression coefficient) AND variation (e.g. standard deviation) or associated estimates of uncertainty (e.g. confidence intervals) |
| ☐ | ☒ | For null hypothesis testing, the test statistic (e.g. $F$, $t$, $r$) with confidence intervals, effect sizes, degrees of freedom and $P$ value noted<br>*Give P values as exact values whenever suitable.* |
| ☒ | ☐ | For Bayesian analysis, information on the choice of priors and Markov chain Monte Carlo settings |
| ☒ | ☐ | For hierarchical and complex designs, identification of the appropriate level for tests and full reporting of outcomes |
| ☐ | ☒ | Estimates of effect sizes (e.g. Cohen's $d$, Pearson's $r$), indicating how they were calculated |

*Our web collection on statistics for biologists contains articles on many of the points above.*

## Software and code

Policy information about availability of computer code

| | |
|---|---|
| Data collection | All software used for data collection in this study was written in house in either Matlab (2017b) or Python (3.7) and OpenCV (4.1). See Methods section. |
| Data analysis | All software used for data analysis in this study was written in house in either Matlab or Python. See Methods section. Software used for model simulations (behavior and Retina models) were made publicly available on Github:<br>https://github.com/harpazone/Modeling-larvae-social-behavior<br>https://github.com/nguyetming/retina_model |

For manuscripts utilizing custom algorithms or software that are central to the research but not yet described in published literature, software must be made available to editors and reviewers. We strongly encourage code deposition in a community repository (e.g. GitHub). See the Nature Research guidelines for submitting code & software for further information.

## Data

Policy information about availability of data

All manuscripts must include a data availability statement. This statement should provide the following information, where applicable:

- Accession codes, unique identifiers, or web links for publicly available datasets
- A list of figures that have associated raw data
- A description of any restrictions on data availability

All raw data used in this manuscript can be found at: https://doi.org/10.7910/DVN/POVJYS

# Field-specific reporting

Please select the one below that is the best fit for your research. If you are not sure, read the appropriate sections before making your selection.

[x] Life sciences [ ] Behavioural & social sciences [ ] Ecological, evolutionary & environmental sciences

For a reference copy of the document with all sections, see nature.com/documents/nr-reporting-summary-flat.pdf

# Life sciences study design

All studies must disclose on these points even when the disclosure is negative.

**Sample size**

For all group swimming experiments we used sample sizes that were large enough to estimate group statistics (e.g. dispersion and alignment) according to previously reported data on collective behavior in zebrafish (see for example Katz et al, 2011, Harpaz et al, 2017 and Methods for statistical procedure and references) and to also allow at least 25 degrees of freedom when parametric statistical models were used to compare between experimental conditions. In the virtual reality assay, we used 40 trials per stimulus as this number proved sufficient to estimate the response of a single fish to the presented stimuli (see also Larsch et al, 2018) and 24-32 fish were used per experiment as our preliminary data showed that these numbers are sufficient to estimate the mean and variance of the responses of the fish to the presented stimuli, and to reliably detect differences between stimuli.

**Data exclusions**

Groups were eliminated from subsequent analysis in the case that one or more of the fish were immobile for more than 25% of the experiment. This criteria was decided based upon preliminary observations, but we have also verified that choosing a more stringent, or a less stringent criteria for elimination did not change the nature of the results. In the VR assay, fish were eliminated and replaced before stimuli presentation begun if they did not show spontaneous swimming for more than 5 minutes.

**Replication**

All experimental results presented here were successfully replicated. We also chose to test two different group sizes (5 and 10 fish in a group) to make sure our results can generalize to groups of different sizes. In the VR assay we show that the the behavioral algorithms we detected and report, also generalized to different stimuli (dots vs ellipses, moving vs. stationary stimuli).

**Randomization**

For all group swimming experiments, fish (of the same age group) were randomly assigned to groups of 5 or 10 fish. In the VR assay, individual fish were randomly assigned to an experiment (see manuscript for different experiments and stimuli). The order of stimuli presentation was randomly shuffled for every fish.

**Blinding**

Experimenters were not blinded to the experimental conditions. For the group swimming experiments, the experimental conditions that we manipulated were age and light vs dark which are directly controlled by the experimenter. For the VR assay, all fish in an experiment were presented with all stimuli and therefore blinding was unnecessary.

# Reporting for specific materials, systems and methods

We require information from authors about some types of materials, experimental systems and methods used in many studies. Here, indicate whether each material, system or method listed is relevant to your study. If you are not sure if a list item applies to your research, read the appropriate section before selecting a response.

## Materials & experimental systems

| n/a | Involved in the study | |
|---|---|---|
| x | | Antibodies |
| x | | Eukaryotic cell lines |
| x | | Palaeontology and archaeology |
| | x | Animals and other organisms |
| x | | Human research participants |
| x | | Clinical data |
| x | | Dual use research of concern |

## Methods

| n/a | Involved in the study | |
|---|---|---|
| x | | ChIP-seq |
| x | | Flow cytometry |
| x | | MRI-based neuroimaging |

## Animals and other organisms

Policy information about studies involving animals; ARRIVE guidelines recommended for reporting animal research

**Laboratory animals**

Zebrafish, Danio rerio of the AB strain at ages 7, 14 and 21 dpf. Sex is not fully determined at these ages.

**Wild animals**

*Provide details on animals observed in or captured in the field; report species, sex and age where possible. Describe how animals were caught and transported and what happened to captive animals after the study (if killed, explain why and describe method; if released, say where and when) OR state that the study did not involve wild animals.*

**Field-collected samples**

*For laboratory work with field-collected samples, describe all relevant parameters such as housing, maintenance, temperature, photoperiod and end-of-experiment protocol OR state that the study did not involve samples collected from the field.*

| | |
|---|---|
| Ethics oversight | All experiments followed institution IACUC protocols as determined by the Harvard University Faculty of Arts and Sciences standing committee on the use of animals in research and teaching |

Note that full information on the approval of the study protocol must also be provided in the manuscript.
